# Assessment of intra-operative bowel perfusion in children using indocyanine green: an exploratory study investigating its effect on surgical management

**DOI:** 10.1007/s00383-026-06432-4

**Published:** 2026-04-27

**Authors:** Jonathan J. Neville, Elizabeth Vincent, Marta Gazzaneo, Michela Marinaro, Anushka Nair, Cecilia Cirelli, Laura Privitera, Paolo De Coppi, Stefano Giuliani

**Affiliations:** 1 Specialist Paediatric and Neonatal Surgery, Great Ormond Street Hospital, London, UK; 2 Great Ormond Street Institute of Child Health, University College London, London, UK

**Keywords:** Paediatrics, Children, Surgery, Indocyanine green, Perfusion, Fluorescence guided surgery

## Abstract

**Background:**

Intra-operative bowel perfusion assessment using indocyanine green (ICG) is an emerging technology that may improve outcomes in paediatric surgery. We aimed to investigate whether intra-operative perfusion assessment using ICG would change a surgeon’s management plan in children undergoing elective stoma closure.

**Methods:**

Four operative videos (two ileostomy and two colostomy closures) showing proximal and distal limbs of bowel immediately prior to anastomosis were independently assessed by paediatric surgeons. Prior to the intravenous injection of ICG, surgeons were asked if perfusion in each stoma limb was ‘adequate’ or ‘poor’, and whether they would complete an anastomosis. After injection of ICG, the same questions were repeated. Primary outcome was the proportion of surgeons who changed their plan after witnessing ICG administration.

**Results:**

In all four stoma closures there were no operative complications and no adverse events related to ICG injection. Twenty-four surgeons participated in the study, resulting in 96 assessments. Twelve (50%) surgeons changed their management plan at least once after witnessing ICG administration. Nineteen surgeons (79%) considered ICG to be useful in this context. Surgeons stated ICG perfusion assessment was useful to confirm their visual assessment of bowel perfusion. Participants highlighted the lack of data linking intra-operative bowel perfusion assessment with ICG to surgical outcomes in children.

**Conclusions:**

Assessment of intra-operative bowel perfusion using ICG resulted in 50% of surgeons changing their management plan. The majority of surgeons believed that ICG was useful in this context. However, the relationship between ICG perfusion and clinical outcomes remains unknown.

## Introduction

Poor intestinal perfusion is a risk factor for complications in gastrointestinal surgery, such as anastomotic leak and stricture formation [1]. In paediatric surgery, the careful evaluation of intra-operative bowel perfusion has an impact on the outcomes of surgery for several conditions, including necrotising enterocolitis (NEC), Hirschsprung’s disease, and reconstructive surgery for anorectal malformations [2, 3]. Traditionally, this assessment has relied on visual and tactile indicators, such as discolouration of the serosa and mucosa, pulsation of vessels in the mesentery, bowel peristalsis and bleeding, which are subjective and challenging to quantify [4]. Indocyanine green fluorescence angiography (ICG-FA) is a novel adjunctive strategy for the intra-operative assessment of bowel perfusion. ICG is an FDA approved water soluble near-infrared cyanine dye with an excellent safety profile [1]. Intravenous ICG remains in the intravascular space, where it has an approximate half-life of 3 min, before it is cleared by the liver. ICG is injected intravenously during surgery, and imaging using a fluorescence camera allows surgeons to assess organ perfusion in real-time. Quantitative ICG-FA adds further objective information based on the characteristics of the ICG fluorescence-time curve. From this, several parameters can be derived, including maximum fluorescence intensity (F-max), time from injection to F-max or half of maximum fluorescence (T-max and T1/2), time to peak (time from the first fluorescence signal to F-max) and the slope (F-max divided by time to peak) [5].

In adult gastrointestinal surgery, several large randomised controlled trials of ICG-FA have been undertaken. These have shown that ICG-FA is a safe and straightforward intra-operative technique which may reduce the incidence of anastomotic leaks when used to guide left-sided colon resections [6]. Despite growing adoption in adult surgery, the use of ICG-FA in paediatric surgery remains limited, and few studies have correlated subjective or quantitative ICG perfusion assessment with clinical outcomes in children undergoing gastrointestinal procedures [1].

Adoption of this technology relies on surgeons changing their management strategy according to the perceived adequacy or inadequacy of perfusion, observed as good or poor ICG-FA signal. Stoma closures are a common elective procedure, relevant to the management of several paediatric surgical conditions, which involve assessment of bowel perfusion intra-operatively. Therefore, the aim of this exploratory study was to investigate whether intra-operative perfusion assessment using ICG-FA would change a surgeon’s management plan in children undergoing elective stoma closure. A secondary explorative aim was to understand the relationship between surgeons’ perfusion assessments with and without ICG, and quantitative perfusion parameters.

## Methods

### Patient cohort and intra-operative videos

**Fig. 1:**
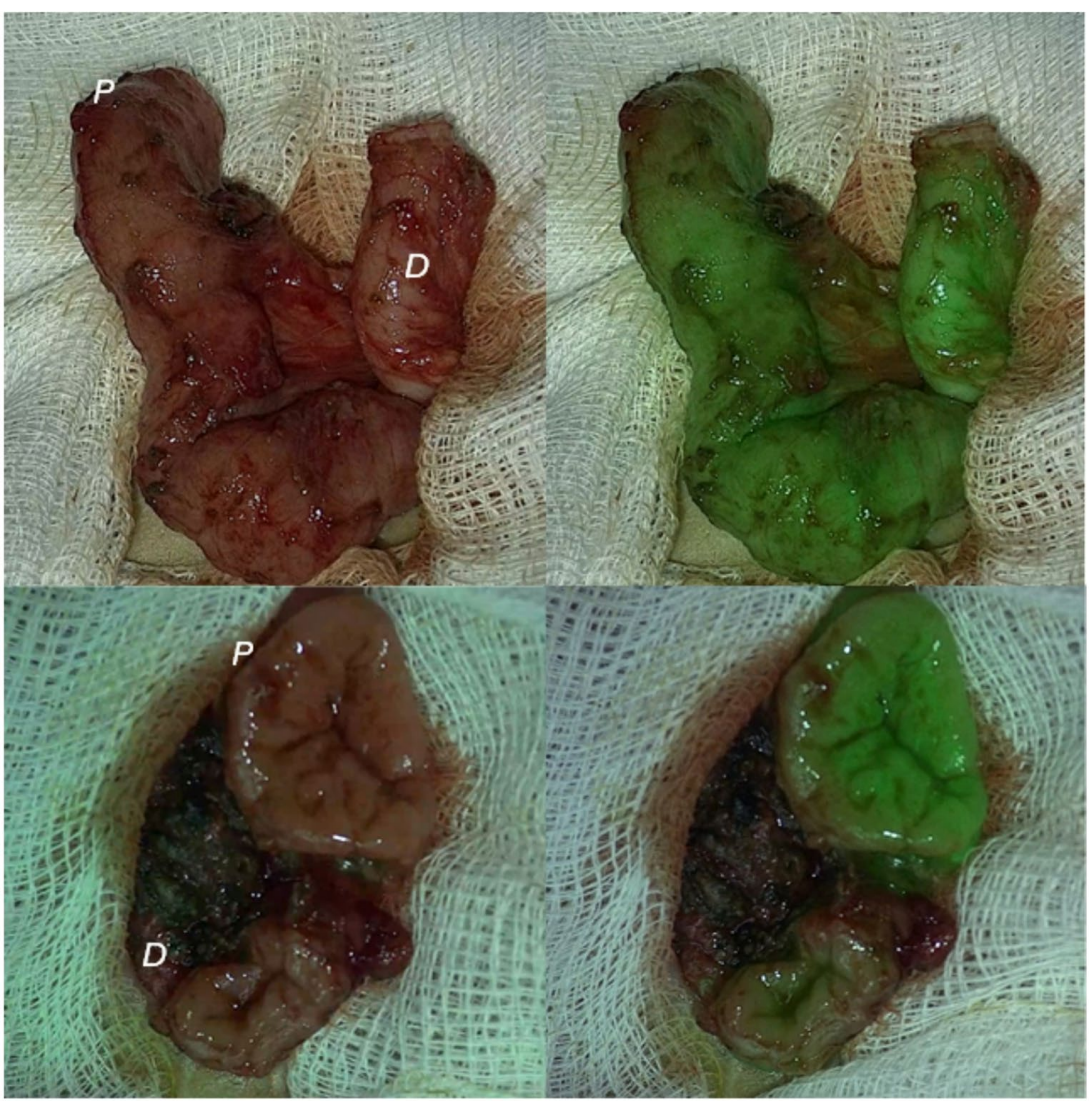
Representative white light (left) and indocyanine green fluorescence angiography (right) images from two cases. The proximal (P) and distal (D) limbs of bowel are shown prior to anastomosis

Standardised intra-operative videos of ICG administration during elective stoma closures were recorded as part of a prospective observational study (enrolled May – December 2024). Two patients undergoing ileostomy closure and two patients undergoing colostomy closure were selected (Table 1). These four videos were chosen because they clearly and consistently showed the proximal and distal loops of bowel before and after ICG injection. All four videos had a uniform positioning of the bowel, with surrounding white gauze. Videos were recorded using a Karl Storz Rubina^®^ lens system (Karl Storz, United Kingdom). After preparation of the bowel for anastomosis, the fluorescent camera was placed 10 cm directly above the operative field (Fig. 1). The video recording was started, and intravenous ICG was administered after 60 s. The ICG dose was 0.01 mg/kg or 0.02 mg/kg, chosen based on the protocol of an ongoing clinical trial (NCT06421103). No standardised protocols for ICG dose exist, however doses 0.01 mg/kg to 0.3 mg/kg have been used within the literature and are considered safe [7]. The recording was continued for 5 min after ICG signal was identified.

**Table 1:** Characteristics of included patients

Case	Age (months)	Sex	Procedure	ICG dose (mg/kg)
1	20	Female	Colostomy closure	0.01
2	15	Female	Ileostomy closure	0.02
3	14	Male	Colostomy closure	0.02
4	16	Female	Ileostomy closure	0.02

*ICG* indocyanine green

ICG fluorescence signal was quantified using PerfusionTech software (PerfusionTech, Copenhagen, Denmark). For each of the four cases, four regions of interest were selected using the software at each of the proximal and distal ends of the bowel. Regions were placed on mucosal or serosal surfaces depending on the orientation of the bowel in the video. For each limb, the mean fluorescence signal (in arbitrary units) was derived from an average of these four regions of interest over time and plotted on a fluorescence-time curve in GraphPad Prism. F-max and slope perfusion parameters were derived from these curves.

### Study procedures

The four pre-recorded videos were independently viewed by a convenience sample of paediatric surgeons who were recruited from two tertiary paediatric surgery centres in the United Kingdom and Italy (March – September 2025). Paediatric surgeons volunteered to participate in each unit, and resident surgeons had a minimum of two years’ experience in the specialty. Intra-operative ICG imaging was routinely used in both centres, however none of the participating surgeons had performed > 40 procedures using bowel ICG-FA. Prior to viewing the recordings, participants were briefed on ICG technology and how to define poor or adequate perfusion based on ICG signal.

Surgeons were first shown the white light images and asked whether they thought the bowel limbs were poorly or adequately perfused, and whether they would be happy to perform an anastomosis. Perfusion was determined by the presence or absence of peristalsis, the colour of the serosa and evidence of bleeding, and relied on the application of the surgeon’s experience. Subsequently, surgeons were shown the bowel after injection of ICG, which occurred during the video recording. The surgeons were then asked if they thought the bowel limbs were poorly or adequately perfused, based on the presence and strength of the ICG fluorescence signal, and whether they would be happy to perform an anastomosis (Fig. 1).

### Study outcomes

The primary outcome of the study was to identify what proportion of surgeons would change their operative plan based on the ICG perfusion assessment. The secondary outcome of the study was to understand ICG utility. Surgeons were also asked whether they believed ICG was helpful in the context. As an explorative aim, we also compared the surgeons’ qualitative assessment of ICG perfusion to the quantitative ICG-FA parameters derived from the fluorescence-time curves.

### Ethical approvals

Ethical approvals to inject ICG and record intra-operative ICG-FA videos for research purposes were obtained (London - Bloomsbury Research Ethics Committee, 23/LO/0743, 7th November 2023). Written informed consent was obtained from each patients’ legal guardians prior to surgery.

### Statistical analysis

Categorical variables were compared via a Chi-squared test. Statistical analysis was performed in SPSS (version 29.0.0). A p-value < 0.05 was considered significant.

## Results

There were no complications from ICG injection in any of the four patients included in this study (Table 1). In all patients an anastomosis was performed without any modification to the operative plan based on ICG perfusion. After 6 months of follow-up, no patients have developed any anastomotic complications.

Twenty-four paediatric surgeons reviewed the four videos, resulting in 96 total non-independent assessments of ICG perfusion (Table 2). Six participants (25%) were consultant surgeons and 18 (75%) were paediatric surgery residents. Overall, the operative management plan was changed in 15/96 (16%) cases. Surgeon seniority did not affect the rate of management plan change (consultants changed their plan in 13% of assessments versus residents in 17%, *p* = 0.626).

**Table 2:** Overview of results

	Case One		Case Two		Case Three		Case Four	
	Proximal	Distal	Proximal	Distal	Proximal	Distal	Proximal	Distal
F-max (A.U.)	49.2	12.0	28.0	26.0	44.6	42.5	42.5	37.5
T-max (seconds)	24.1	24.7	34.6	35.3	15.2	20.1	17.3	25.4
Slope (A.U./second)	2.0	0.5	0.8	0.7	2.9	2.1	2.5	1.5
Frequency of surgeons grading perfusion as adequate with white light	23 (96%)	17 (71%)	24 (100%)	22 (92%)	16 (66%)	24 (100%)	24 (100%)	20 (83%)
Frequency of surgeons grading perfusion as adequate post-ICG	24 (100%)	10 (42%)	24 (100%)	22 (92%)	24 (100%)	23 (96%)	24 (100%)	21 (88%)
Frequency of surgeons changing management	7 (29%)		2 (8%)		5 (21%)		1 (4%)	

*A.U.* arbitrary units, *ICG* indocyanine green, *F-max* maximum fluorescence signal, *T-max* time in seconds from ICG injection to maximum fluorescence signal, *slope* maximum fluorescence signal divided by the time from the start of fluorescence signal to maximum fluorescence

Of the 24 surgeons surveyed, 12/24 (50%) changed their plan at least once after witnessing ICG injection. In nine cases, surgeons who had planned to perform a bowel anastomosis changed to not performing an anastomosis after observing ICG-FA. In six cases, surgeons who had stated that they would not perform an anastomosis changed their decision to perform an anastomosis after witnessing ICG-FA.

**Fig. 2:**
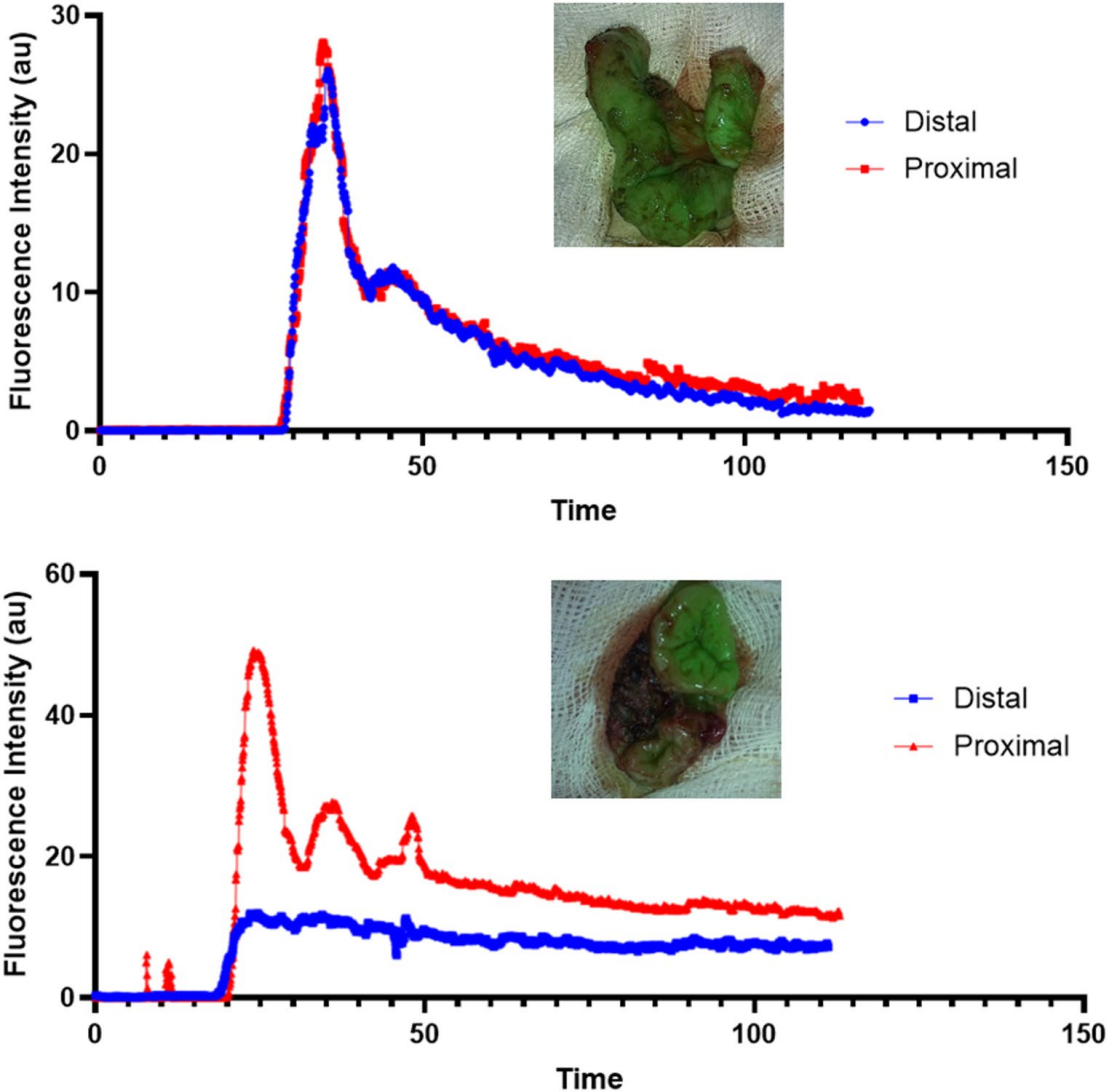
Representative indocyanine green fluorescence angiography images and mean fluorescence intensity-time (seconds) curves. The cases illustrate similar (top) and differential (bottom) fluorescence intensities and associated quantitative metrics in the distal and proximal stoma limbs. *AU* arbitrary units

Eight ICG fluorescence-time curves were plotted, each corresponding to a single proximal or distal limb of bowel (Fig. 2). Parameters from the fluorescence-time curves were compared to the proportion of surgeons who changed their management plan in each patient (Table 2). In case one, there was a four-fold difference in F-max and slope between the proximal and distal stoma limbs. In this case, 29% of surgeons changed their management plan. In case three, 33% of surgeons believed perfusion was poor in the proximal limb of bowel with white light images. After ICG-FA, 100% of surgeons rated perfusion in the proximal limb as adequate. Here, the F-max and slope parameters were similar in the proximal and distal limbs. The difference in F-max and slope between the proximal and distal stoma limbs was less pronounced in the remaining two cases. In these cases, the management plan was altered by only 8% and 4% of surgeons respectively. These cases highlight how surgeons can change their management plan with qualitative assessments of ICG-FA and how the assessment may associate with quantitative parameters.

Nineteen (79%) surgeons believed that ICG was helpful in the context of elective stoma closure. Positive feedback focused on how ICG-FA supported decision making, by confirming adequate intestinal perfusion, thereby increasing confidence in a surgeon’s decision. However, the paucity of normal reference signal information and patient outcome data were highlighted as potential pitfalls.

## Discussion

In this study, we have demonstrated that ICG-FA may be beneficial in supporting intra-operative bowel perfusion assessment in children. Overall, half of surgeons considered changing their management plan at least once after administration of ICG. Surgeon seniority did not have a significant impact on the rate of management change, although this comparison was likely underpowered. The majority of surgeons considered ICG-FA useful in this clinical context.

Studies investigating surgeon assessment of ICG-FA have shown high inter-observer variability. Larsen et al. found that surgeons’ assessment of 40–43 pre-recorded ICG-FA videos of low anterior resections resulted in poor agreement in time to fluorescence signal visibility, time to F-max, and time to disappearance of ICG signal [8]. Based on these parameters, surgeons were unable to differentiate between patients who did and did not develop an anastomotic leak. However, on quantitative analysis, the ICG slope was significantly lower in patients that developed an anastomotic leak, suggesting poor arterial flow into the bowel. Hardy et al. compared surgeons who were experienced with ICG-FA against surgeons who were not experienced, and identified that agreement was greater between expert surgeons when assessing colon perfusion [9]. Similarly, Nerup et al. observed that quantitative ICG-FA greatly improved the decision making of surgeons inexperienced at using ICG [10]. Together these results highlight the potential pitfalls of qualitative ICG-FA perfusion assessment. Assessment of > 50 cases may be required to obtain the experience needed to reliably assess perfusion using qualitative ICG-FA [11]. These studies highlight the learning curve associated with ICG-FA, and suggest that combining ICG-FA assessment with quantitative analysis may improve assessment of bowel perfusion, especially for surgeons inexperienced in its use. In this study, we observed surgeons altering their management plans in two directions: deciding against performing an anastomosis that was initially planned, and opting to perform an anastomosis when they previously stated they would not. This may indicate that ICG-FA provides greater clarity, but may also suggest that ICG increases uncertainty, especially in surgeons less experienced with ICG-FA. In paediatric surgery, training programmes may be required to disseminate ICG-FA best practice and shorten the learning curve. Studies are also required that include paediatric surgeons with extensive experience using ICG-FA.

In paediatric gastrointestinal surgery the use of ICG-FA for bowel perfusion assessment is in its early stages. Yada et al. reported the use of ICG-FA during two colostomy closures in paediatric patients [12]. In both cases, ICG signal was observed 20–30 s after injection at the resection margins, and both anastomoses were performed without complications. ICG-FA has also been used to assess colonic, rectal and vaginal tissue perfusion during pull-through surgery for Hirschsprung’s disease (three patients), and reconstructive surgery for anorectal malformations (one patient) and cloaca (nine patients) [3]. Here ICG-FA resulted in a change in management plan in four (31%) cases. In three of these cases, the level of bowel used for the reconstruction was changed to a more proximal section (> 10 cm higher), and in one case the distal bowel was discarded and the colostomy was used for the pull through. Meisner et al. used ICG to assess the perfusion of 55 oesophageal anastomoses in 53 children [13]. They reported that qualitative ICG perfusion scores were significantly lower in patients that developed anastomotic complications. Recently, Zhang et al. published their experience utilising quantitative ICG-FA to assess the anastomosis site in transanal pull-through for Hirschsprung’s disease [14]. Thirty-four children underwent ICG-FA, compared to visual inspection in 133. There was no significant difference in the incidence of anastomotic complications between the two groups, but the study was likely underpowered. To date, no complications from the use of ICG for bowel perfusion assessment have been reported in children [1, 15].

Quantitative ICG-FA parameters, derived from fluorescence-time curves, may provide surgeons with an objective measure of bowel perfusion. The F-max represents the peak fluorescence intensity achieved, and higher values indicate greater perfusion. Similarly, the slope (rate of increase in fluorescence signal) is an indicator of adequate tissue perfusion. A low slope suggests reduced blood inflow. However, larger studies in children that correlate quantitative ICG-FA metrics with histopathology and clinical outcomes are required to define age-specific values for F-max and slope in normal and hypoperfused bowel. Dalloul et al. compared quantitative ICG-FA analysis strategies in a segment of paediatric colon that contained areas pre-determined to have adequate and poor perfusion by white light assessment [16]. They identified specific usability and applicability limitations with all analysis approaches used, which highlight the challenges of translating quantitative ICG-FA into clinical practice. Reproducible computational approaches that are developed for the operative environment are required to generate reliable and interpretable quantitative ICG-FA perfusion data that can guide surgical decision-making.

Evidence from randomised control trials in adult gastrointestinal surgery have shown that ICG-FA in robotic or laparoscopic colonic resections does not significantly reduce the rates of anastomotic leaks [6, 17, 18]. However, studies suggest that ICG-FA assessment may be beneficial in left-sided colonic surgery and in total mesorectal excision of rectal cancer [6, 19–21]. A systematic review and meta-analysis of 27 cohort and randomised studies comparing ICG-FA to standard care in colorectal anastomosis reported that ICG-FA was significantly associated with a lower probability of anastomotic leak and complications [22]. Across these studies the weighted mean rate of change in surgical plan was 9.6% (range: 0.6–28.8%), suggesting considerable variability in the interpretation of ICG-FA signal. Interestingly, a change in management plan with ICG-FA was associated with an increased risk of an anastomotic leak.

This study is limited by the use of pre-recorded videos. As such, surgeons were unable to dynamically inspect and palpate the bowel as they would be able to in a normal procedure. This may limit the real-world comparability of the white light assessment and result in an incorrect assessment of bowel perfusion. The study was also conducted in a simulated environment, devoid of the typical operative pressure, which may impact the decision-making process and limit the transferability of the results. Multiple ICG doses were used, limiting comparison between patients and preventing formal statistical comparisons between quantitative and qualitative assessments. The quantitative ICG-FA metrics are therefore descriptive and hypothesis generating, and should be interpreted with caution. Lower doses were utilised as part of an ongoing study investigating lower ICG doses in paediatric surgery (NCT06421103). We selected stoma closures as it is a reproducible and representative procedure involving bowel perfusion assessment, however, the generalisability of the findings of this study to other procedures is limited. The majority of surgeons in the study were relatively inexperienced in the use of ICG-FA, and therefore the findings from this study may not be relevant to more experienced surgeons. No patients developed an anastomotic complication and therefore no conclusions can be drawn regarding white light or ICG-FA assessments and clinical outcomes.

This study is predominantly exploratory in nature, with a descriptive outcome. As such, further work is needed to understand the role of ICG-FA in paediatric surgery. In order to do this, multicentre studies are required which correlate ICG-FA qualitative and quantitative features with clinical outcomes in children undergoing specific paediatric gastrointestinal surgical procedures. Initially, research should focus on determining the utility of ICG-FA in procedures where assessment of bowel perfusion is critical for outcomes, such as oesophageal atresia repair, surgery for NEC, or reconstructive surgery for Hirschsprung’s disease or anorectal malformations. Study endpoints must utilise key surgical outcomes, such as anastomotic leak rate, anastomotic stricture rate, and development of short gut syndrome in NEC.

In conclusion, we have shown that ICG-FA is a safe and feasible tool to assess bowel perfusion during elective paediatric surgery, and that it has the potential to change surgeons’ management in a proportion of cases. Definitive adoption of ICG-FA into routine clinical practice should be guided by evidence that it improves surgical outcomes. As such, further work must investigate the impact of ICG-FA bowel perfusion assessment on outcomes in children in multicentre prospective trials. Implementation will require standardised training programmes, robust quantitative analysis methods, and validated references ranges in children.

## Data Availability

No datasets were generated or analysed during the current study.
